# Association of Sarcopenia with and Efficacy of Anti-PD-1/PD-L1 Therapy in Non-Small-Cell Lung Cancer

**DOI:** 10.3390/jcm8040450

**Published:** 2019-04-03

**Authors:** Naoya Nishioka, Junji Uchino, Soichi Hirai, Yuki Katayama, Akihiro Yoshimura, Naoko Okura, Keiko Tanimura, Sachi Harita, Tatsuya Imabayashi, Yusuke Chihara, Nobuyo Tamiya, Yoshiko Kaneko, Tadaaki Yamada, Koichi Takayama

**Affiliations:** Department of Pulmonary Medicine, Graduate School of Medical Science, Kyoto Prefectural University of Medicine, Kyoto 602-8566, Japan; g4h4n93w@koto.kpu-m.ac.jp (N.N.); hirasoh9@koto.kpu-m.ac.jp (S.H.); ktym2487@koto.kpu-m.ac.jp (Y.K.); aki-y@koto.kpu-m.ac.jp (A.Y.); ku-n07@koto.kpu-m.ac.jp (N.O.); keiko-t@koto.kpu-m.ac.jp (K.T.); harita@koto.kpu-m.ac.jp (S.H.); imabayas@koto.kpu-m.ac.jp (T.I.); c1981311@koto.kpu-m.ac.jp (Y.C.); koma@koto.kpu-m.ac.jp (N.T.); kaneko-y@koto.kpu-m.ac.jp (Y.K.); tayamada@koto.kpu-m.ac.jp (T.Y.); takayama@koto.kpu-m.ac.jp (K.T.)

**Keywords:** sarcopenia, non-small cell lung cancer, psoas major muscle area, red blood cell distribution width

## Abstract

Secondary sarcopenia is defined as a decrease in muscle mass due to disease or malnutrition. Several studies have reported that secondary sarcopenia is an indicator of postoperative recurrence. We hypothesized that there is a correlation between the effect of immune checkpoint inhibitors (ICIs) and sarcopenia. We retrospectively analyzed 38 patients with advanced non-small cell lung cancer (NSCLC) who were treated with ICIs between February 2016 and April 2018. Patients were divided into two groups according to the change rate of the psoas major muscle area (PMMA) at the L2–L3 position and investigated the correlation between the change rate of the PMMA and the efficacy of ICIs was investigated. The objective response and disease control rates were lower in patients with sarcopenia than in those without sarcopenia. Patients with sarcopenia exhibited a significantly shorter median progression-free survival (PFS) than non-sarcopenia patients. Moreover, focusing on good Eastern Cooperative Oncology Group performance status patients, sarcopenia patients showed a shorter PFS than non-sarcopenia patients. Patients with sarcopenia are associated with poor outcomes for immunotherapy among those with advanced NSCLC, based on retrospective analysis. Further research is needed to validate the clinical biomarkers involved in ICI responders.

## 1. Introduction

As cancer progresses, cancer causes malnutrition with various metabolic disorders and exacerbates the patient’s prognosis and quality of life. We call this state cachexia. Unlike normal malnutrition, cachexia often causes decreases in skeletal muscle mass from an early stage in a phenomenon defined as sarcopenia [1,2]. Sarcopenia is mainly caused by aging, but disease or malnutrition can also cause significant decreases in muscle, which is defined as secondary sarcopenia.

To evaluate sarcopenia-associated deterioration, physical findings including weight change, clinical symptoms, and biochemical examinations have been used as clinical indicators. However, in recent years, CT images and MRI tomographic images have been used as methods to calculate decreases in muscle mass and are highly evaluated for objectivity and accuracy [3]. Moreover, as an evaluation method of sarcopenia other than imaging, Kim et al. suggested red cell distribution width (RDW), which is usually used to determine variability in the size of circulating erythrocytes [4]. They reported that RDW might reflect the existence of chronic inflammation, which is one of the major causes of sarcopenia.

Immune checkpoint inhibitors (ICIs) have received great attention since their appearance and are expected to significantly improve prognosis. However, patients who can receive successful ICI therapy are limited, comprising only 20% of the total [5]. Therefore, many researchers have been exploring the cause of the insufficient effect of ICI.

It has recently been reported that a poor Eastern Cooperative Oncology Group performance status (ECOG PS) is strongly related with poor progression-free survival (PFS) [6,7]. Based on this fact, we believe that sarcopenia may be associated with the therapeutic effect of ICI. Therefore, using the psoas major muscle area (PMMA) at the L2–L3 position, we evaluated the change rate of PMMA between the CT at the first visit and the CT before ICI administration. In addition to studying PMMA, the specific outcomes of treatment were recorded and their relationship to the PMMA change was investigated. Further, we examined whether it could be used as a biomarker in ICI therapy.

## 2. Experimental Section

### 2.1. Patients

In a retrospective cohort study, we evaluated all Japanese patients with previously treated advanced non-small cell lung cancer (NSCLC) who were administered either nivolumab or pembrolizumab at the hospital of Kyoto Prefectural University of Medicine between January 2016 and May 2018. The primary objective was to investigate the association between clinical outcomes and the PMMA change rate. The study was approved by the Clinical Research Ethics Committee of University Hospital, Kyoto Prefectural University of Medicine. The following showed the eligibility criteria: (1) There was prior treatment with one or more chemotherapy before ICI treatment, (2) patients were defined as stage III or stage IV according to the TNM staging (seventh edition), (3) patients had measurable disease lesions, (4) patients underwent a CT or PET/CT within four weeks before ICI treatment, and (5) the CT included the patient’s psoas major muscle at the L2–L3 position.

We collected the following data for all eligible patients from their charts: age, sex, stage, ECOG PS at the time of ICI initiation, serum albumin level and CRP (before ICI administration), RDW (before ICI administration), smoking status, histology, molecular profiling for EGFR and ALK when available, PD-L1 when available, previous treatment, PMMA measured by two physicians, response status, date of progression as decided by CT, date of death, and the last follow-up.

### 2.2. Tumor Genomic Analysis

We investigated the EGFR mutation and ALK translocation for all adenocarcinoma and non-squamous non-small cell lung cancer cases. EGFR mutations were detected by the Cobas EGFR mutation test (Roche Molecular Systems, Pleasanton, CA) with exon 18–21 sequencing conducted at commercial clinical laboratories (SRL, Inc. and BML, Inc., Tokyo, Japan).

### 2.3. Tumor PD-L1 Analysis

After obtaining insurance approval in Japan, we investigated PD-L1 expression using the IHC 22C3 pharmDx assay or 28-8 pharmDx assay and analyzed at SRL, Inc., as allowable. A tumor proportion score was calculated by the pathologists of the commercial vendor.

### 2.4. Definition of Psoas Major Muscle Area

**Figure 1:**
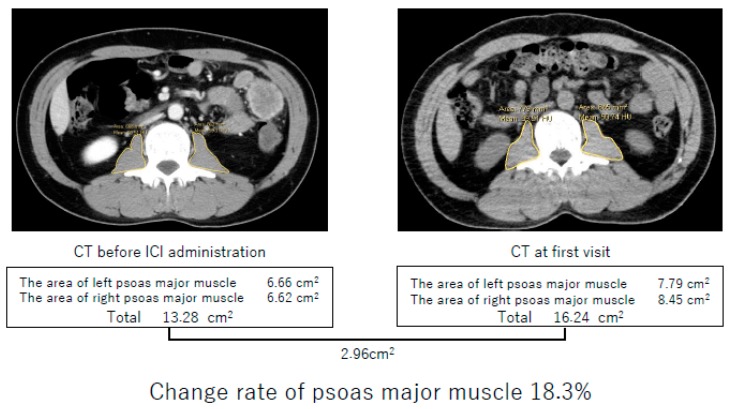
Method of calculating change rate of the psoas major muscle area (PMMA). PMMA was calculated as the sum of both the right and left area of the psoas major muscle at the L2–L3 position. In this case, PMMA before immune checkpoint inhibitor administration was 13.28 cm^2^, and PMMA at first visit was 16.24 cm^2^. Using these data, we could calculate the change rate of PMMA: Change rate of PMMA (%) = (1 − 13.28/16.24) × 100 = 18.3.

We defined the primary exposure variable as the change rate of PMMA. PMMA was calculated as the sum of both the right and left area of the psoas major muscle at the L2–L3 position. These areas were measured in the region of interest by tracing an outline, using the image viewer software “DICOM” (Figure 1). The change rate of PMMA was defined as follows:

Change rate of PMMA (%) = (1 − PMMA before ICI start/PMMA at diagnosis) × 100

Yaguchi et al. reported that the change rate of PMMA in the recurrence group was higher than that in the no recurrence group; they also reported that the evaluation of the change rate of PMMA after gastrectomy could assist in the diagnosis of cancer progression [8]. Similar to the average rate of change of PMMA of each group shown by Yaguchi et al., we found that the change rate of PMMA in the no recurrence group maintained within 10% and assumed that more than a 10% change of PMMA might be associated with tumor progression or relapse. This phenomenon could be related to cancer immunity. Therefore, we dichotomized patients into the change rate of PMMA ≥10% (sarcopenia) group or <10% (non-sarcopenia) group. After dichotomizing patients, we evaluated clinical outcomes of ICI between these two groups.

### 2.5. Endpoints

Progression free survival was defined as the number of days between the administration of the first immune checkpoint inhibitor (either nivolumab or pembrolizumab) and progression or death, whichever occurred earlier. Overall response rate (ORR) was counted as the sum of the complete response and partial response as per the RECIST guidelines, and disease control rate (DCR) was defined as the sum of the complete response, partial response, and stable disease as specified in the RECIST guidelines.

### 2.6. Statistical Analyses

The Mann-Whitney *U* test and Fisher’s exact test were used to compare groups and categorical variables. PFS was estimated using the Kaplan-Meier method and compared by the log-rank test. Correlation between RDW and the rate of change of PMMA was estimated using Spearman’s rank correlation analysis. Statistical analyses were performed using EZR. A *p* value of <0.05 was considered statistically significant [9].

## 3. Results

### 3.1. Patient Characteristics

The baseline characteristics of the enrolled patients are listed in Table 1. Patients consisted of 26 males (68%) and 12 females (32%) in this group. The median age at the initiation of the nivolumab or pembrolizumab treatment was 68.76 years (range, 46–85 years; interquartile range, 64.5–72). Patients with ECOG PS less than one accounted for 74% of patients and those with PS more than two accounted for 26% of patients. The median change rate of PMMA was 13% (range, 7%–30%), with a change rate of <10% in 17 patients (45%) and ≥10% in 21 patients (55%).

Twenty-seven patients with non-squamous histology were investigated for ALK translocation and EGFR mutation. None of them had ALK translocation, and six patients (22%) had an EGFR mutation. Of all 38 patients, 23 patients (61%) had PD-L1 testing on tumor samples.

Interval of CT measurement between the time of first visit and around 4 weeks of ICI administration was 311.5 days (range, 74–2850 days).

### 3.2. Psoas Major Muscle Area (PMMA)

**Figure 2:**
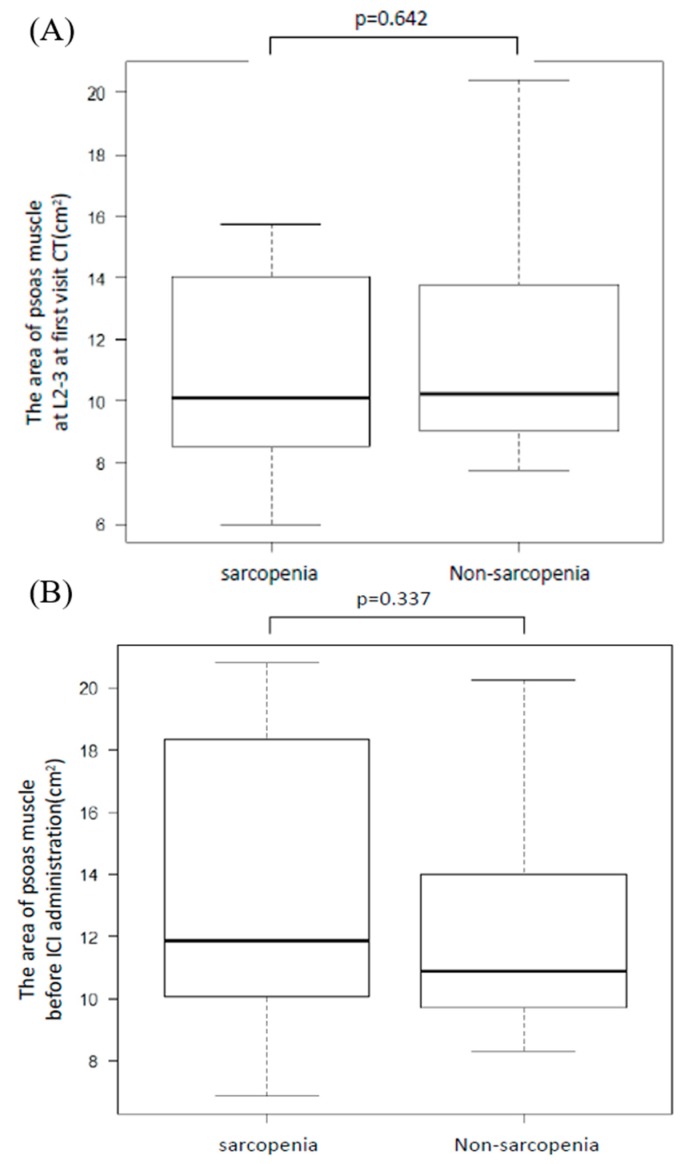
Area of psoas major muscle at the L2–L3 position for the sarcopenia and non-sarcopenia groups. We divided patients into sarcopenia or non-sarcopenia groups and compared the area of the psoas major muscle. There was no significant difference at the first visit CT (**A**) or before ICI treatment (**B**).

**Figure 3:**
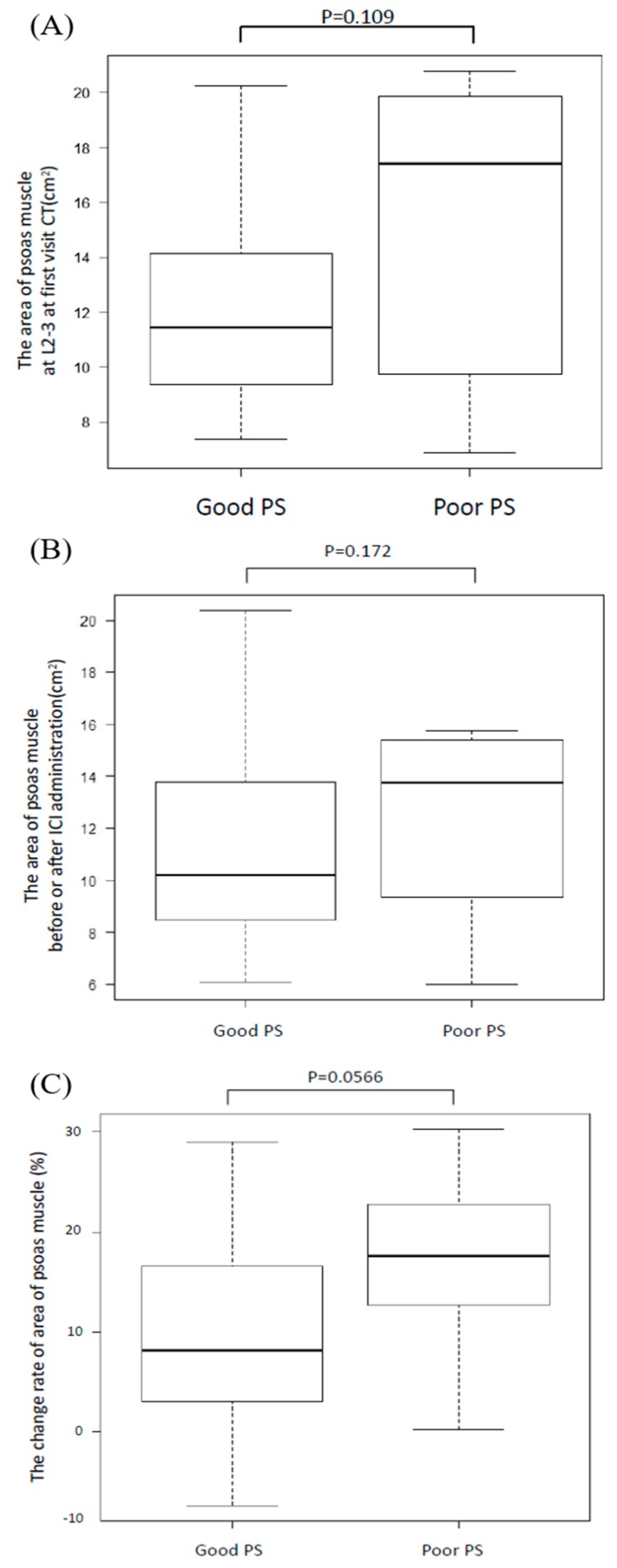
Area of psoas major muscle at the L2–L3 position for the good PS and poor PS groups. We divided patients into good PS (less than one) or poor PS groups (more than two) and compared their psoas major muscle areas (PMMAs). There was no significant difference at the first visit CT (**A**) or before ICI treatment (**B**). Moreover, we evaluated the change rate of area of psoas muscle between good PS patients and poor PS patients. It has turned out that poor PS group tended to be higher the change rate than good PS group (**C**).

We evaluated PMMA with a CT taken at the time of first visit and within 4 weeks before ICI administration and the change rate of PMMA between the sarcopenia and non-sarcopenia groups. While we defined sarcopenia as a change rate more than 10%, no difference was found in PMMA between these groups regardless of the CT measurement time (*p* = 0.642, *p* = 0.337, Figure 2A,B, respectively). Moreover, we divided all patients into good PS (PS = 0 or 1 before ICI administration) or poor PS groups (PS is more than two before ICI administration) and measured PMMA (Figure 3A,B). There was no significant difference in PMMA, but the change rate was higher in the poor PS group (*p* = 0.056, Figure 3C).

### 3.3. Clinical Outcome

We analyzed the ORR and DCR in the sarcopenia and non-sarcopenia groups (Table 2). The ORR and DCR were significantly lower in the sarcopenia group than the non-sarcopenia group (0% versus 41% (*p* = 0.0154) and 24% versus 58% (*p* = 0.0458)).

**Figure 4:**
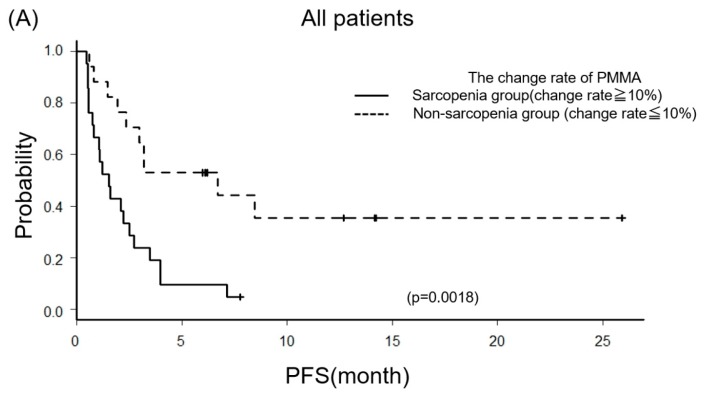

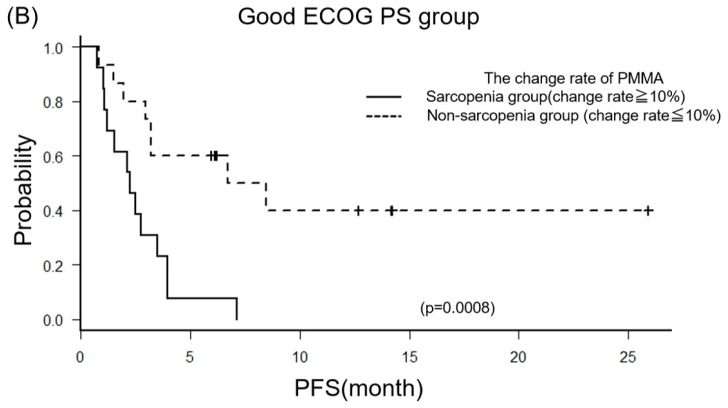
Kaplan–Meier curves of the progression-free survival (PFS). We compared PFS between sarcopenia and non-sarcopenia groups, including all patients (**A**) or focusing on the good PS group (**B**).

The PFS was estimated and displayed in Figure 4A. PFS of the sarcopenia group was significantly shorter than the non-sarcopenia group (Median 47 days (95% CI: 23–76) versus 204 days (95% CI: 59–NA) (*p* = 0.00186)). Moreover, we evaluated the PFS between the sarcopenia and non-sarcopenia groups, with a focus on the good PS group (Figure 4B). Similarly, the sarcopenia group was significantly shorter than the non-sarcopenia group (Median 45 days (95% CI: 18–NA) versus 230 days (95% CI: 59–NA) (*p* = 0.00404)).

### 3.4. Red Cell Distribution Width (RDW) and Sarcopenia

**Figure 5:**
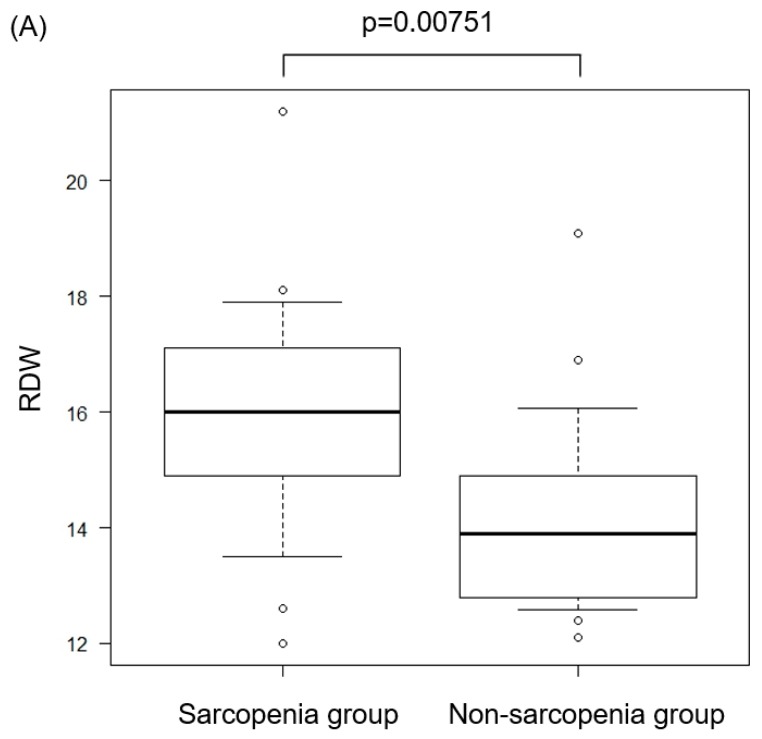

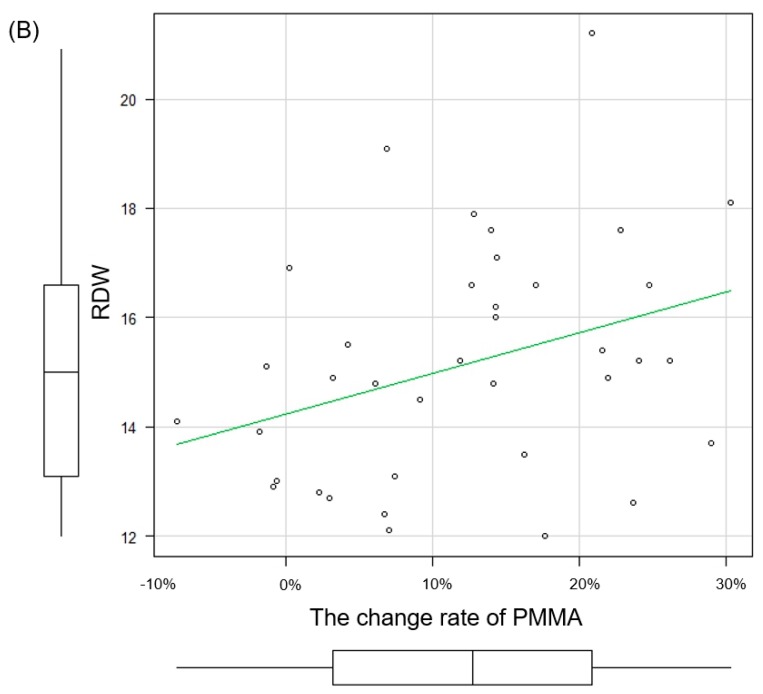
Red cell distribution width (RDW) and sarcopenia. We compared RDW between sarcopenia and non-sarcopenia groups (**A**). Moreover, we evaluated the correlation coefficient by the Spearman’s rank correlation between the change rate of PMMA and RDW (**B**).

RDW is one of the parameters of the complete blood count (CBC). This parameter evaluates variation in red blood cell size or volume. RDW is a quantified value of the variability of the size of circulating erythrocytes and is calculated as the standard deviation of the distribution of red blood cell size divided by the MCV [10]. This value is routinely reported as part of the CBC. We evaluated RDW between sarcopenia and non-sarcopenia groups. The RDW value of the sarcopenia group was significantly higher than the non-sarcopenia group (*p* = 0.00751, Figure 5A). Further, in the Spearman’s correlation analysis, there was a degree of correlation between the change rate of PMMA and RDW (*r* = 0.337, *p* = 0.0387, Figure 5B).

## 4. Discussion

It is known that treatment with ICI may result in dramatic therapeutic effects for cancer patients; however, most patients cannot receive this attractive advantage. Turner et al. and Flint et al. reported that the metabolic abnormality caused by cachexia progression is a major component [11,12].

Cachexia is defined as a multifactorial syndrome characterized by the irreversible loss of skeletal muscle mass. To diagnose cancer cachexia, one of the following three points needs to be satisfied: 1) A weight loss >5% over the past 6 months (in the absence of simple starvation); 2) BMI <20 and any degree of weight loss >2%; or 3) appendicular skeletal muscle index consistent with sarcopenia and any degree of weight loss >2% [13]. Sarcopenia included in the cachexia criteria is defined as a decrease in muscle mass, and this term was defined by Rosenberg [14]. Sarcopenia is largely divided into two types. One is known as primary sarcopenia caused by aging, and the other is secondary sarcopenia caused by cancer or other diseases [15]. In this study, sarcopenia represented secondary sarcopenia.

Many articles have reported about sarcopenia and its evaluation [16,17]. One of them is the body mass index (BMI). Frequently, patients with sarcopenia tend to have low BMI; however, it is recognized that some patients do not adhere to this criterion [18,19]. For example, sarcopenic obesity is a condition of the body in which the muscle has been reduced; however, excess fat is attached. Therefore, despite the decrease in muscle mass, sarcopenic obese patients tend to have a higher apparent BMI. As a result, BMI has not been considered a reliable criterion in recent years [20,21].

When evaluating muscle mass with images, dual energy X-ray absorptiometry, CT, and MRI have been used. According to the European Working Group on Sarcopenia in Old People, CT is the simplest way to evaluate skeletal muscle among them [15,22,23]. CT is most widely used for evaluation, and especially the PMMA at the L2–L3 position is often used as an indicator.

Many studies have reported that both psoas muscle index (PMI), which is PMMA divided by the body surface area, and skeletal muscle mass index (SMI), which is the skeletal muscle area divided by the body surface area, have been very useful for predicting patients’ prognosis [24]. However, although cut-off values were set in each article, the values were different in each, and utility was not so high.

In recent years, the usefulness of the neutrophil-to-lymphocyte ratio (NLR) as an ICI biomarker has been revealed [25,26]. Certainly, NLR is an inexpensive and readily available biomarker, which provides additional prognostic information to identify patients benefiting from ICI treatment, but there is no report that NLR is independent of PS. As a result, there is a possibility that this variable may correlate with PS. Since this NLR eventually reflects the total inflammation/immunity state and the number of lymphocytes shows the nutritional status, it is likely that PS decreases as the number of lymphocytes decreases (NLR rises). Therefore, further research is needed to investigate the relationship between NLR and PS in order to discuss the effectivity of NLR.

In this study, we focused on the change rate of PMMA at the L2–L3 position as an indicator of sarcopenia and explored the correlation between sarcopenia and the ICI response. Yaguchi et al. reported that the change rate of PMMA in the non-recurrence group after gastrectomy was significantly less than that in the relapsed group [8]. Their article also showed the average change rate of PMMA of each group, and we recognized by their figures that the boundary of the average change rate of PMMA between the two groups appeared to be 10% [8]. Therefore, based on this report, we set the cut-off value to 10% in this study. Patients were dichotomized into the change rate of PMMA <10% (non-sarcopenia group) or ≥10% (sarcopenia group). After dichotomizing patients, we found that the sarcopenia group had a significantly shorter PFS than the non-sarcopenia group (Figure 4A). Moreover, we next selected only patients with a good PS from all patients and dichotomized them into two groups. We compared the PFS and found that the PFS of the sarcopenia group was also significantly shorter than the others (Figure 4B). From this result, we found that decreasing PMMA by more than 10% might prevent patients from gaining the ICI therapeutic effect, even if patients had a good PS. In that sense, it is more useful than NLR.

Chronic inflammation is one of the causes of sarcopenia affecting immunotherapy. Causes of immunosuppression include (1) an increase of immunosuppressive cells and (2) a suppression of antitumor T cell activity by immunocompetent molecule binding. Immunosuppression induced by chronic inflammation is caused by an increase in immunosuppressive cells in the tumor locus. In particular, myeloid-derived suppressor cells (MDSCs) are strongly associated with immunosuppression, and induction of MDSCs into tumor cells by chronic inflammation not only suppresses the action of CD8+ T cells in the tumor tissue, it can also induce regulatory T cells (T reg) to suppress immune responses [27,28,29,30].

As a result, since anti-PD-1/PD-L1 inhibitors can only inhibit immune suppression through the immunity checkpoint, increased MDSCs and Tregs by chronic inflammation cannot prevent tumor immunity itself within the microenvironment. This is thought to have a great influence on the therapeutic effect [27,28,29,30,31].

In addition, red blood cell distribution width (RDW) has attracted attention recently as a marker of chronic inflammation. Although it has not been clarified which mechanism is related between RDW and chronic inflammation, a large cohort study suggested that RDW is a strong marker of inflammatory activity, such as hs-CRP and ESR [32].

Further, Kim et al. has previously proven that RDW has a positive correlation with sarcopenia, and our data also showed a significant increase in RDW in the sarcopenia group when comparing sarcopenia and non-sarcopenia groups (Figure 5A) [4].

Moreover, considering the correlation between the PMMA change rate and RDW, sarcopenia and chronic inflammation are strongly related, and the effect of immunotherapy is hindered by the persistent chronic inflammation causing sarcopenia (Figure 5B).

There are several limitations in this study. First, this study is a retrospective study with a small sample size. Therefore, there is a potential for bias and confounding factors. Second, the change rate of PMMA is affected by the patient’s PS. In the case of patients with a poor PS, even if their change rates of PMMA are less than 10%, we could not expect successful therapeutic effects of ICI. This reason is that these patients may already be in a sarcopenic state at their first visit, and their psoas major muscle may have atrophied. If patients are in a state of sarcopenia at their first visit, further atrophy of their PMMA is not likely to occur, and the change rate of PMMA tends to be lower. Third, half of the patients had not performed PD-L1 testing; therefore, we could not consider the effect of PD-L1. Lastly, we could not evaluate the patient’s aging effect. In this study, the interval of CT from the first visit to ICI administration was about eight years at maximum, and it is not yet known how much PMMA changes in healthy subjects by aging.

## 5. Conclusions

Based on a retrospective analysis, patients with sarcopenia were associated with poor outcomes for treatment with immunotherapy among those with advanced NSCLC, and it is a possibility that this poor outcome may be caused by chronic inflammation. Further research is needed to validate the clinical biomarkers involved in ICI responders.

## Figures and Tables

**Table 1:** The baseline characteristics of the patients.

	Overall	The Rate of Change of Psoas Major Muscle	
	(*n* = 38)	≥10%	<10%
		(*n* = 21)	(*n* = 17)
Age (years)			
Median (range)	68.7 (46–85)	67.1 (46–82)	70.8 (55–85)
Sex			
Male Female	26 12	15 6	11 6
ECOG PS			
0–1 ≥2	28 10	13 8	15 2
Pathology			
Squamous Non-squamous	11 27	6 15	5 12
Clinical stage			
III IV Postoperative recurrence	12 20 6	4 16 1	8 4 5
PD-L1			
≥50% 1–49% 0% unknown	7 9 7 15	4 5 3 9	3 4 4 6
Treatment line			
Second line therapy More than third line therapy	19 19	10 11	9 8
Driver mutations			
EGFR ALK	6 0	4 0	2 0
Interval of CT measurement			
Median days (range)	311.5 (74–2850)	299 (102–2850)	352 (74–2154)

**Table 2:** Overall response rate (ORR) and disease control rate (DCR) in the sarcopenia and non-sarcopenia groups.

	Sarcopenia Group	Non-Sarcopenia Group	*p* Value
ORR	0 of 21 (0%)	7 of 17 (41%)	0.0154
DCR	5 of 21 (24%)	10 of 17 (58%)	0.0458
